# Role of Selective Serotonin Reuptake Inhibitors in the Treatment of Hypochondriasis: A Systematic Review

**DOI:** 10.7759/cureus.45286

**Published:** 2023-09-15

**Authors:** Manoj Prithviraj, Palani Selvam Mohanraj, Tejas K Patel, Arani Das

**Affiliations:** 1 Psychiatry, All India Institute of Medical Sciences, Gorakhpur, IND; 2 Biochemistry, All India Institute of Medical Sciences, Gorakhpur, IND; 3 Pharmacology, All India Institute of Medical Sciences, Gorakhpur, IND; 4 Physiology, All India Institute of Medical Sciences, Gorakhpur, IND

**Keywords:** selective serotonin reuptake inhibitors, hypochondriacal symptoms, health anxiety, ssri, illness anxiety, hypochondriasis

## Abstract

Hypochondriasis is a condition characterized by an unrealistic fear of having a serious medical illness resulting in health anxiety. Currently, no evidence-based pharmacological treatment options are available for the treatment of hypochondriasis. Since selective serotonin reuptake inhibitors (SSRIs) are the first-line treatment option for anxiety disorders, they may be useful for relieving hypochondriasis symptoms. Moreover, off-label use of SSRIs in these cases is highly prevalent in clinical practice. Thus, in this study, we aimed to review the available literature to assess the role of SSRIs in the treatment of hypochondriacal symptoms. A systematic search was conducted in PubMed, Scopus, ScienceDirect, Embase, and Cochrane Database of Systematic Reviews from the date of inception to December 2022. We included only randomized clinical trials (RCTs) investigating the effect of SSRIs in the treatment of hypochondriacal symptoms. Non-RCTs, observation studies, and animal studies were excluded. The Risk of Bias 2 tool was used to assess the quality of included studies. Out of 2264 articles, six RCTs met our inclusion criteria. Studies have been conducted using different SSRIs in the treatment of primary hypochondriasis and hypochondriacal symptoms associated with other psychiatric disorders. All the studies have found that the use of SSRIs has some beneficial role in improving hypochondriacal symptoms. This suggests that SSRIs may be one of the promising pharmacological interventions in the treatment of hypochondriasis.

## Introduction and background

Hypochondriasis is a chronic debilitating condition characterized by severe health anxiety. According to the International Classification of Diseases 11th Revision (ICD-11), it is defined as a state of having excessive fear about having a life-threatening and progressive disease accompanied by maladaptive health behaviors [1]. In the Diagnostic and Statistical Manual of Mental Disorders, 5th Edition (DSM-5), it has been replaced by the term illness anxiety disorder [2]. It is highly prevalent in general healthcare settings but often goes unnoticed or missed. A study by Tyrer et al. across five different specialty clinics found that 19.8% of the study population had significant health anxiety [3]. The patient suffers persistent anxiety and worries due to fear of having a severe physical problem even after a series of normal investigations and reassurance from the medical professionals. Minor physical symptoms can be interpreted as a major ailment, which can lead to recurrent visits to doctors and unwarranted clinical investigations. Intrusive thoughts and preoccupation about physical health cause frequent palpitations, nervousness, muscle tension, tremors, sweating, and various other unexplained somatic complaints [4]. These symptoms of autonomic overactivity can be again perceived as signs of some undiagnosed and grave illness leading to a vicious cycle. Unrecognized hypochondriasis can negatively impact the financial costs of medical care and also strain the available health infrastructure [5]. It also causes impairment in the personal, social, and occupational life of the individual resulting in severe distress. Psychotherapies including cognitive behavior therapy, mindfulness, and interpersonal therapy have been useful in alleviating anxiety symptoms [6-8]. However, the role of pharmacotherapy in treating hypochondriasis is not well studied. Hypochondriasis can occur comorbid with other psychiatric disorders like obsessive-compulsive disorder (OCD), panic disorder, and major depressive disorder (MDD) [9]. A study by Van den Heuvel et al. has demonstrated that patients with hypochondriasis show alterations in the frontal-striatal brain regions during functional magnetic resonance imaging(fMRI) similar to OCD [10]. Since antidepressants are effective in the management of other anxiety spectrum disorders, they may be considered as one of the treatment options for hypochondriasis [11]. In the current literature, reports are available about the use of selective serotonin reuptake inhibitors (SSRIs), serotonin noradrenaline reuptake inhibitors (SNRI), tricyclic antidepressants (TCA), pregabalin, and gabapentin in the management of patients with hypochondriasis [12]. Though the exact mechanism is unclear, it is proposed that antidepressants could relieve pain symptoms and exert a calming effect on the patients, thus minimizing health-seeking behavior. On the contrary, at times use of antidepressants to treat hypochondriasis may be counterproductive as medicines may not be working the same way in every patient and the side effects of medications can be mistaken as a sign of illness and lead to more illness anxiety [13,14]. Hence, the role of antidepressants in hypochondriasis is still debatable due to the lack of definite evidence. Since serotonin reuptake inhibitors are generally the first-line agents to be used in other anxiety disorders [15], this systematic review aimed to assess the efficacy and utilization of SSRIs in the treatment of hypochondriasis. The objective was to evaluate the efficacy and use of SSRIs in the treatment of hypochondriasis.

## Review

Methods

This systematic review was conducted following the Preferred Reporting Items for Systematic Reviews and Meta-Analyses (PRISMA) guidelines for reporting systematic reviews [16]. The study population included patients diagnosed with hypochondriasis based on DSM or ICD guidelines, or those who were treated for symptoms of hypochondriasis as part of other disorders, regardless of age or gender, and in any treatment setting. The intervention being evaluated was the use of SSRIs for symptoms of hypochondriasis, with the comparison being patients who were treated with a placebo, other medications, behavioral therapy, psychotherapy, or other relevant treatment strategies. The primary outcome measures were changes in the severity of hypochondriasis symptoms as assessed by standard scales such as the Yale-Brown Obsessive-Compulsive Scale for Hypochondriasis (H-YBOCS), Whiteley Index (WI), Health Anxiety Inventory (HAI), Heightened Illness Concern (HIC) severity scale, Illness Attitude Scale, Columbia Heightened Illness - Obsessive Compulsive Scale (CHIC-OCS), and other related self- and clinician-reported measures. Additional outcome measures included changes in the severity of other psychopathologies such as anxiety, depression, obsession-compulsive behavior, attitude toward illness, and quality of life, as well as the number of participants who experienced adverse events and serious adverse events in both the intervention and control groups. The review protocol was registered on the International Prospective Register of Systematic Reviews (PROSPERO) with the registration ID CRD42020197019.

Search Strategy

A systematic literature search was conducted in PubMed, Scopus, ScienceDirect, Embase, and Cochrane Database of Systematic Reviews from the date of inception to May 2022, as well as searching the bibliographies of relevant research articles. Only studies in the English language were included. We used the following search strategy as described in Table 1.

**Table 1:** Search strategy

Database	Search terms
PubMed	"hypochondriasis"[MeSH Terms] OR "hypochondria*"[All Fields] OR ("hypochondriacal"[Title/Abstract]) OR "hypochondriacal neuros*"[Title/Abstract] OR "illness anxiety"[All Fields] OR "health anxiety"[All Fields] AND ("serotonin uptake inhibitors"[MeSH Terms] OR ("serotonin"[All Fields] AND "uptake"[Title/Abstract] AND "inhibitors"[Title/Abstract]) OR "serotonin uptake inhibitors"[Title/Abstract] OR "ssri*"[Title/Abstract] OR "selective serotonin reuptake inhibitor*"[All Fields] OR "serotonin reuptake inhibitor"[All Fields] OR ("selective"[Title/Abstract] AND "serotonin"[Title/Abstract] AND "reuptake"[Title/Abstract] AND "inhibitor*"[Title/Abstract]) OR ("sertraline"[All Fields] OR "sertraline"[MeSH Terms]) OR ("paroxetine"[All Fields] OR "paroxetine"[MeSH Terms]) OR ("fluoxetine"[MeSH Terms] OR "fluoxetine"[All Fields]) OR ("fluvoxamine"[All Fields] OR "fluvoxamine"[MeSH Terms]) OR ("citalopram"[MeSH Terms] OR "citalopram"[All Fields] OR "escitalopram"[All Fields]) OR ("drug"[Title/Abstract] AND "therapy"[Title/Abstract]) OR "drug therapy"[Title/Abstract] OR ("pharmacological"[Title/Abstract] AND "treatment"[Title/Abstract]) OR "pharmacological treatment"[Title/Abstract])
Scopus	(ALL (hypochondriasis OR "Illness anxiety" OR "Health Anxiety" OR "Hypochondriacal" OR "Hypochondria*" )) AND (ALL (ssri OR "Selective Serotonin reuptake inhibitors" OR fluoxetine OR fluvoxamine OR sertraline OR paroxetine OR citalopram OR escitalopram )) AND ( LIMIT-TO (DOCTYPE, "ar") ORLIMIT-TO (DOCTYPE, "re"))
ScienceDirect	(Hypochondriasis OR "Illness anxiety" OR "Health Anxiety" OR "Hypochondriacal" OR "Hypochondria" OR ''Hypochondriac'') AND (''Selective Serotonin reuptake inhibitors'')
Cochrane Central Register of Controlled Trials	(Hypochondriasis OR "Illness anxiety" OR "Health Anxiety" OR "Hypochondriacal" OR "Hypochondria" OR ''Hypochondriac'') AND (SSRI OR "Selective Serotonin reuptake inhibitors" OR Fluoxetine OR Fluvoxamine OR Sertraline OR Paroxetine OR Citalopram OR Escitalopram)
Embase	Hypochondriasis [Emtree] AND Serotonin uptake inhibitors [Emtree]

Selection Procedure

This systematic review included randomized controlled trials (RCTs) that evaluated the use of SSRIs in the treatment of primary hypochondriasis or symptoms of hypochondriasis that occur as part of other psychiatric disorders. Studies that were excluded from this review included observational studies, case reports, editorials, commentaries, review articles, and animal studies. The screening and selection process of articles was carried out using an open-access online tool called CADIMA version 2.2.3. (Julius Kühn Institute, Quedlinburg, Germany). To ensure objectivity, the screening process of articles was performed by two independent authors, and any disagreement was resolved by a third author. Initially, the articles were screened based on their title and abstract, and then the full text of the selected articles was screened for inclusion in the review. The data collection from the included studies was done independently by two authors.

Quality Assessment

**Figure 1:**
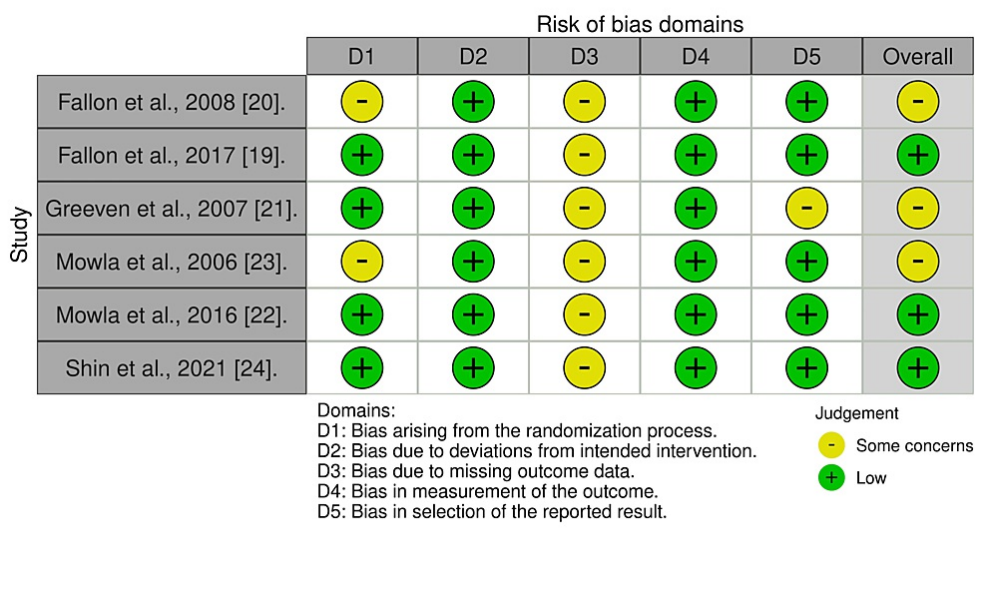
Risk of bias summary

**Figure 2:**
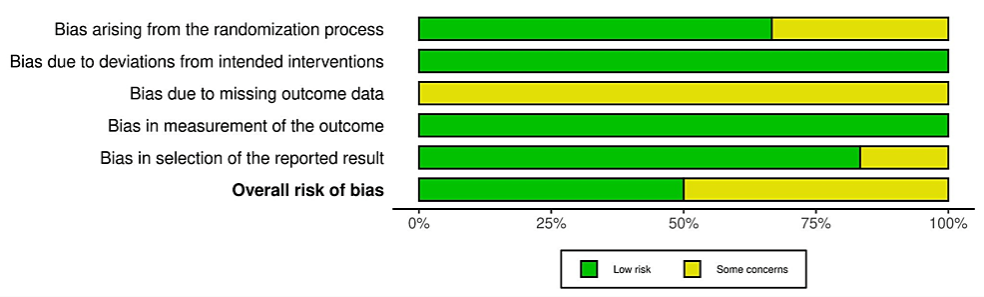
Risk of bias graph

The quality of the included studies was evaluated by using the Cochrane tool to assess the risk of bias in RCTs [17]. The studies were categorized into three groups: high risk of bias, low risk of bias, and unclear risk of bias. The risk of bias summary and graph were created using the online tool Risk-of-bias VISualization [18] and the summary of studies is shown in Figures 1, 2.

Results

**Figure 3:**
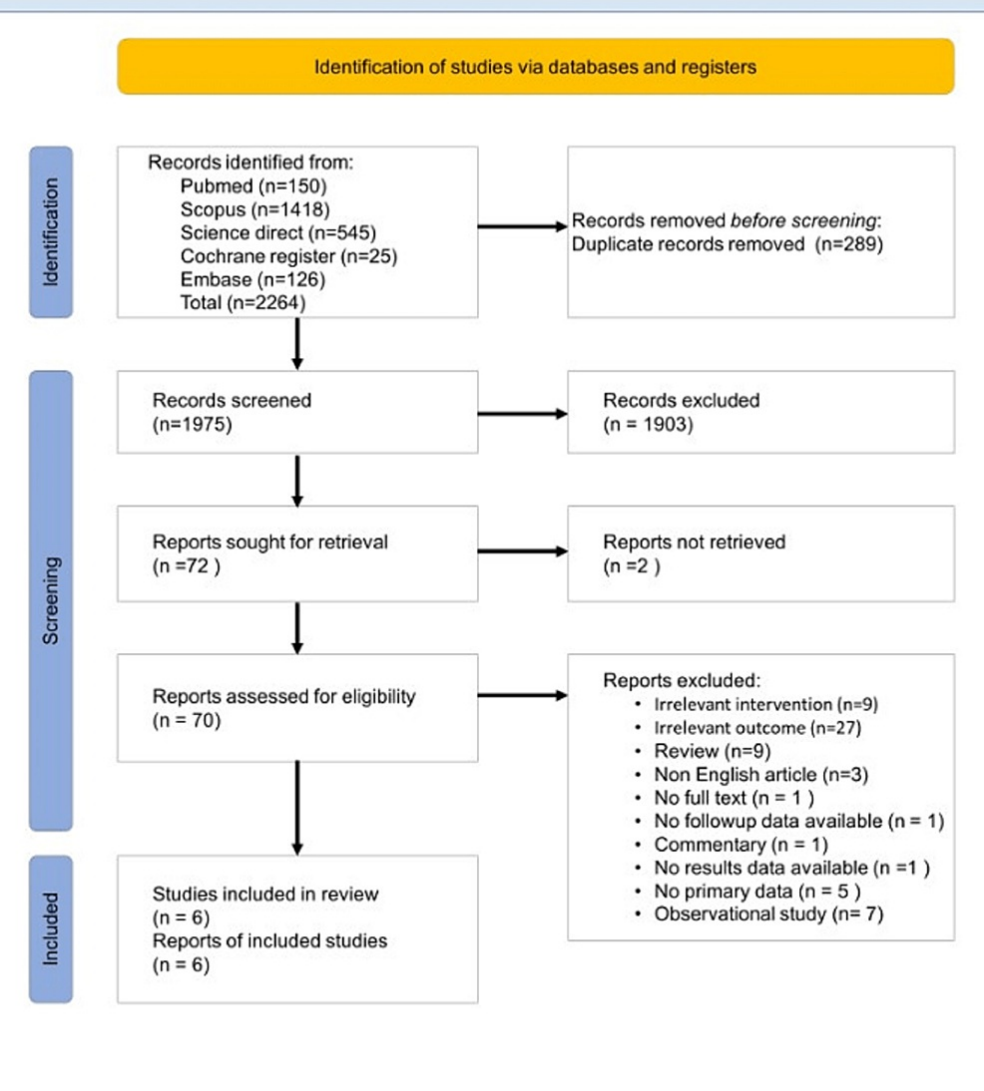
PRISMA flow diagram PRISMA: Preferred Reporting Items for Systematic Reviews and Meta-Analyses.

The search strategy for this systematic review yielded a total of 1975 articles after removing duplicates and merging results from multiple databases. After screening the titles and abstracts, 72 articles were eligible for full-text screening, and six studies were finally included in the review. PRISMA flow chart of the search process is shown in Figure 3.

Study Characteristics

The studies were conducted in the USA [19,20], the Netherlands [21], Iran [22,23], and South Korea [24]. The subjects included in the study were patients with primary hypochondriasis and hypochondriacal symptoms in MDD. SSRIs used include fluoxetine [19,23], paroxetine [21], sertraline [22], escitalopram, and vortioxetine (Table 2) [24]. The sample size of these studies ranged from 45 to 195 subjects with the duration of intervention with SSRI ranging from six to 24 weeks. The comparison group varied between studies and included placebo, cognitive behavior therapy (CBT), or other drugs (Table 3).

**Table 2:** Basic characteristics of included studies * Data are presented as mean ± SD or median (range). RCT: randomized control trial; C: comparator group; SSRI: selective serotonin reuptake inhibitor; MDD: major depressive disorder; FXT: fluoxetine; PXT: paroxetine; SER: sertraline; EST: escitalopram; VXT: vortioxetine; NTP: nortriptyline; DXT: duloxetine; DFX: desvenlafaxine; CBT: cognitive behavior therapy; P: placebo.

Reference	Country	Population	SSRI used	Sample size, total (SSRI/C)	Age in years*	Gender distribution, M/F	Design
Fallon et al. (2008) [20]	USA	Hypochondriasis	Fluoxetine	45 (24 FXT/21 P)	37.8 ± 9.2	12M/12F	RCT
Fallon et al. (2017) [19]	USA	Hypochondriasis	Fluoxetine	195 (45 FXT/44P/53 CBT/53 FXT+CBT)	39.7 ± 14.3	20M/25F	RCT
Greeven et al. (2007) [21]	The Netherlands	Hypochondriasis	Paroxetine	112 (37 PXT/40 CBT/35 P)	41.3 ± 11.7	16M/21F	RCT
Mowla et al. (2006) [23]	Iran	MDD	Fluoxetine	56 (36 FXT/20 NTP)	30.8 ± 9.9	8M/18F	RCT
Mowla et al. (2016) [22]	Iran	MDD	Sertraline	63 (32 SER/31 DXT)	41.2 (19-56)	22M/32F	RCT
Shin et al. (2023) [24]	South Korea	MDD	Escitalopram and vortioxetine	124 (42 EST/42 VXT/40 DFX)	41.4 ± 16.1	28M/96F	RCT

**Table 3:** Details of intervention used CBT: cognitive behavior therapy.

Reference	SSRI used	Dosage	Duration	Comparison group
Fallon et al. (2008) [20]	Fluoxetine	20-80 mg/day	12 weeks	Placebo
Fallon et al. (2017) [19]	Fluoxetine	10-80 mg/day	24 weeks	Placebo/CBT/fluoxetine + CBT
Greeven et al. (2007) [21]	Paroxetine	10-60 mg/day	16 weeks	CBT/placebo
Mowla et al. (2006) [23]	Fluoxetine	40 mg/day	8 weeks	Nortriptyline
Mowla et al. (2016) [22]	Sertraline	50-200 mg/day	6 weeks	Duloxetine
Shin et al. (2023) [24]	Escitalopram, vortioxetine	10-20 mg/day, 10-20 mg/day	6 weeks	Desvenlafaxine

The primary outcome measure of these studies was the hypochondriasis score, which was evaluated using different scales such as WI, HIC severity scale, CHIC-OCS, H-YBOCS, Illness Attitude Scale, and Hamilton Depression Scale (HAM-D). All included studies showed improvement in symptom severity and a decrease in hypochondriasis scores after treatment with SSRIs. The common side effects of SSRIs include gastrointestinal distress, tachycardia, sexual dysfunction, dizziness, headache, body pain, fatigue, loss of appetite, insomnia, dry mouth, and somnolence (Table 4).

**Table 4:** Study outcome and main findings IAS: Illness Attitude Scale; HIC: Heightened Illness Concern; CHIC-OCS: Columbia Heightened Illness Concern–Obsessive-Compulsive Scale; HAM-D: Hamilton Depression Scale; H-YBOCS-M: Hypochondriasis – Yale-Brown Obsessive Compulsive Scale – Modified.

Reference	Measures of hypochondriasis used	Outcome	Adverse effects
Fallon et al. (2008) [20]	Whiteley Index, HIC severity scale, CHIC-OCS	Fluoxetine treatment improved hypochondriasis	Gastrointestinal distress and tachycardia
Fallon et al. (2017) [19]	H-YBOCS-M, Whiteley Index	Fluoxetine treatment improved hypochondriasis	Details not available
Greeven et al. (2007) [21]	Whiteley Index, IAS	Paroxetine treatment improved hypochondriasis	Sexual dysfunction, dizziness, headache, body pain, fatigue
Mowla et al. (2006) [23]	Hypochondriasis subdomain of HAM-D scale	Fluoxetine treatment improved hypochondriacal symptoms	Details not available
Mowla et al. (2016) [22]	Hypochondriasis subdomain of HAM-D scale	Sertraline treatment improved hypochondriacal symptoms	Loss of appetite, gastric disturbances, sexual problems
Shin et al. (2023) [24]	Hypochondriasis subdomain of HAM-D scale	Escitalopram and vortioxetine treatment improved hypochondriacal symptoms	Fatigue, insomnia, dry mouth, somnolence

Discussion

SSRIs are the first-line agents in the treatment of anxiety disorders. Off-label use of SSRIs in hypochondriasis has been highly prevalent in clinical practice. In this review, we investigated the available literature for RCTs that examined the role of SSRIs in the treatment of hypochondriasis. We found six RCTs using common SSRIs such as fluoxetine, paroxetine, sertraline, escitalopram, and vortioxetine. The effectiveness and the clinical utility of individual drugs will be discussed separately in the following section.

Fluoxetine

Out of six RCTs included in this review, three studies examined the role of fluoxetine in the treatment of hypochondriasis. Among them, two studies were conducted in patients with primary hypochondriasis. In a study by Fallon et al. (2008), fluoxetine was found to be moderately efficacious, well tolerated, and beneficial in improving hypochondriasis symptoms in the acute as well as maintenance phase compared to placebo on various commonly used hypochondriasis assessment scales [20]. In another RCT by Fallon et al. (2017), monotherapy with fluoxetine alone was found to be superior in the reduction of hypochondriasis symptoms compared to placebo and combined treatment with CBT and this clinical improvement was independent of comorbid psychiatric disorder. In this study, fluoxetine was more effective with a rapid reduction of symptoms when used in higher doses(40-80 mg) [19]. Based on this RCT, Fallon et al. (2021) have done an exploratory study, which found that participants with high somatic symptoms may be more likely to respond to fluoxetine than CBT in patients with hypochondriasis. This shows that somatic symptoms in hypochondriasis may be a predictor for response to fluoxetine therapy [25]. A similar observation was seen in a previous open-label study where a higher dose (60-80 mg) of fluoxetine was more effective in reducing hypochondriacal symptoms [26]. One possible explanation could be since hypochondriacal symptoms are often obsessive in nature, higher doses of SSRI may be required similar to the treatment of OCD. In both these RCTs, fluoxetine was well tolerated by the participants making it one of the safe molecules for the treatment of hypochondriasis. In the study done by Mowla et al. (2006) among patients with MDD, scores on the hypochondriasis subdomain had significantly improved when treated with fluoxetine compared to nortriptyline [23]. Fluoxetine being one of the first-line treatment options for other anxiety disorders, treatment with the same can be helpful in hypochondriasis too. In an open-label trial, Demopulos et al. reported a similar observation, where eight weeks of treatment with fluoxetine was found to significantly decrease hypochondriacal symptoms in depressed patients [27]. In another study by Fallon et al. (1993), fluoxetine was also effective in reducing the symptoms of hypochondriasis in patients without depression [26]. Based on this available literature, fluoxetine appears to be one of the good pharmacological options in the treatment of hypochondriasis. However, this conclusion has to be viewed with caution as the treatment period was only 12-24 weeks within a small sample of patients. Till now, only one long-term follow-up study is available, which states that SSRI treatment with fluvoxamine and fluoxetine is effective and significantly predicts a positive outcome in hypochondriasis [28]. One probable factor may be pharmacological treatment with SSRI reduces anxiety symptoms, which may have an encouraging role in improving the patient’s compliance toward other non-pharmacological strategies.

Paroxetine

In this review, we found only one RCT that evaluated the use of paroxetine in the treatment of hypochondriasis. In that study, Greeven et al. investigated the treatment of hypochondriasis with CBT and paroxetine compared with a placebo. They have reported that there was a reduction in the severity of hypochondriacal symptoms measured by the WI in both paroxetine and CBT but there was no significant difference between the groups [21].

In another study by the same author, a naturalistic follow-up of the same study participants was done for a period of 18 months. In the follow-up period, hypochondriacal symptoms were measured by the WI at one, five, and 18 months. They concluded the initial treatment effect of CBT and paroxetine was sustained during the follow-up period but did not reveal any significant differences in the course of symptom amelioration between CBT and paroxetine [29]. This result is in accordance with another study by Oosterbaan et al., who have also reported amelioration of hypochondriacal thoughts and behavior measured by Maastrichter Eigen Gezondheids-Attitude en Hypochondrie-schaal (MEGAH) and H-YBOCS-M score after 12 weeks of paroxetine treatment [30]. The findings of these studies suggest that paroxetine may have a positive effect in improving hypochondriacal symptoms. Paroxetine is commonly used for panic attacks and anxiety disorders due to its predominant sedative properties because of its anticholinergic, antihistaminic, and antiadrenergic effects. It has the most potent serotonin reuptake inhibition among the SSRI group [31]. This unique property of paroxetine to act on multiple neurotransmitter receptors may be useful for improving hypochondriacal symptoms.

Sertraline

Sertraline is one of the widely used SSRIs in the treatment of major psychiatric disorders such as anxiety spectrum disorders, depression, and OCD. Few studies have investigated the use of sertraline in hypochondriasis, and overall, the results have been positive. A study included in this review by Mowla et al. (2016), compared the effects of sertraline and duloxetine on signs and symptoms of MDD. This RCT included 63 patients, who were randomly assigned to receive either duloxetine or sertraline for a period of six weeks. Patients in the sertraline group had a significant reduction in hypochondriasis symptoms compared to those in the duloxetine group [22]. This finding is consistent with the results of an open-label trial conducted by Kipper et al. (2019), who investigated the effect of sertraline treatment on personality traits in patients with panic disorder. After treatment with sertraline, there was a significant decrease in Minnesota Multiphasic Personality Inventory scale scores, indicating an improvement in overall personality traits [32]. In both of these studies, sertraline was found to show improvements in the hypochondriasis subdomain. Overall, these studies suggest that sertraline may be effective in treating hypochondriasis symptoms, whether they occur as part of panic disorder or MDD. However, further research is needed to better understand the mechanisms underlying these effects and to determine the optimal dosages and treatment durations for this population.

Other SSRIs in Hypochondriasis

Fluvoxamine: In our extensive literature search, we have found two studies investigating the effect of fluvoxamine in the treatment of hypochondriasis. In an open-label trial by Fallon et al. (2003), fluvoxamine had shown improvement in the severity of hypochondriacal symptoms after 10 weeks [33]. In another study by Schweitzer et al. (2011), a long-term follow-up with combined therapy of fluvoxamine and fluoxetine was shown to improve hypochondriasis symptoms [28]. They concluded that the SSRI use in follow-up was a significant predictor of good prognosis in these patients. Though these preliminary findings showed positive outcomes but lack of a randomized double-blind, placebo-controlled study was a limitation.

Escitalopram: Escitalopram has the most selective serotonergic reuptake inhibition among the SSRI group [34]. In this review, only one study was found in which escitalopram has been shown to improve the hypochondriacal subdomain of HAM-D scores among patients with anxious depression. In the same study, vortioxetine also was found to improve the symptoms of hypochondriasis but the difference was not statistically significant. However, in an open-label study by Park et al., escitalopram was not found to have any effect on the hypochondriacal subdomain of the HAM-D scale in depression patients [35]. In the absence of studies among patients with primary hypochondriasis, sufficient evidence is not available to comment on the effect of escitalopram in patients with hypochondriasis.

## Conclusions

Till now, there is no standard pharmacological option available for the treatment of hypochondriasis. SSRIs are commonly used as an adjuvant along with psychotherapy to relieve anxiety and depression in patients with hypochondriacal symptoms; however, their efficacy in reducing the severity of hypochondriasis was unclear. All the RCTs included in this review have concluded that SSRIs are effective in reducing symptom severity across the commonly used assessment scales for hypochondriasis. This finding has been supported by other open-label trials in which SSRI was found to be effective in the treatment of hypochondriacal symptoms. Based on these observations, SSRIs can be considered one of the promising treatment options in the management of hypochondriasis.
